# An Empirical Analysis of Popular Press Claims Regarding Linguistic Change in President Donald J. Trump

**DOI:** 10.3389/fpsyg.2018.02311

**Published:** 2018-11-21

**Authors:** Marc N. Coutanche, John P. Paulus

**Affiliations:** ^1^Department of Psychology, University of Pittsburgh, Pittsburgh, PA, United States; ^2^Learning Research and Development Center, University of Pittsburgh, Pittsburgh, PA, United States; ^3^Brain Institute, University of Pittsburgh, Pittsburgh, PA, United States

**Keywords:** linguistics, language, cognition, linguistic change, president, Trump

## Abstract

Linguistic features of a person’s speech can change over time. It has been proposed that characteristics in the speech of President Donald J. Trump (DJT) have changed across time, though this claim has been based on subjective and anecdotal reports. A previous study of speech by Presidents of the United States identified an increase in the use of conversational fillers and non-specific nouns, and lower unique word counts, in the speech of President Ronald W. Reagan, but not in the speech of President George H.W. Bush. To empirically test claims of a systematic change in speech by DJT, we applied the same analysis by transcribing and analyzing publicly available Fox News interviews with DJT between 2011 and 2017. A regression analysis revealed a significant increase in the use of filler words by DJT over time. There was no significant change in numbers of unique words. The observed rise in filler words was significantly greater than filler-word change in President George H.W. Bush, and was not significantly different from the rise previously found in the speech of President Ronald W. Reagan. Identifying the reason for this linguistic change is not possible from speech samples alone, and the variables index linguistic change rather than being validated measures of change in cognitive ability. Nonetheless, features of the data such as the trajectory starting years before announcement of candidacy rule-out several potential explanations. To summarize, we find statistical evidence to support suggestions that speech by DJT has changed over time.

## Introduction

There has been speculation that the speech of the current President of the United States, Donald J. Trump (DJT), has systematically changed over time (e.g., Begley, 2017; Pierce, 2017). Anecdotal accounts, subjective judgments, and single comparisons of speech samples have been used to support or refute this suggestion. A more systematic and objective analysis of this question is possible by examining unscripted TV interviews with DJT. In this paper, we analyze TV interviews to systematically address this question.

When a person’s linguistic system is in demand, such as when responding to questions, a failure to compensate can be apparent in the person’s speech, including using over-learned words and phrases (Kemper et al., 2001; Berisha et al., 2015) and filler words (Christenfeld, 1994). Here we examine linguistic markers that are sensitive to this and have comparable reference points: namely one example of linguistic decline and one example of linguistic stability, in responses to questions about current affairs. For example, Berisha et al. (2015) reported an analysis of unscripted speech in news conferences by Presidents Ronald Wilson Reagan and George Herbert Walker Bush (Berisha et al., 2015) to test the possibility that President Reagan experienced linguistic decline during his time in office (Gottschalk et al., 1988). A longitudinal statistical analysis showed that unscripted speech by President Reagan, but not President Bush, had increasing numbers of conversational fillers (“um,” “uh,” etc.) and non-specific (NS) nouns (e.g., “something”), with fewer unique words. In the case of President Reagan, this linguistic decline was hypothesized to relate to a subsequent diagnosis of Alzheimer’s Disease, though such changes can occur for a number of reasons (including aging; Horton et al., 2010).

In this study, we draw on 7 years of television interviews by DJT to test for linguistic change using measures that were previously applied to the speech of presidents (Berisha et al., 2015). Unlike other scales, these measures come from similar contexts, namely unscripted public responses to publicly broadcast questions about current affairs, which differ from other speech in a number of respects, such as awareness that responses are broadcast, a focus on complex topics, and others. A comparison with two presidents responding in comparable contexts (with one showing linguistic change and one not) allows linguistic features to be assessed on an equivalent scale. It should be noted that these variables index linguistic change, but have not been validated as measures of change in cognitive ability so should not be interpreted as such - identifying the reason for any linguistic change is a separate question that is not definitively answerable from speech samples alone. For instance, individuals differ in how they respond to advanced age, which in turn can be reflected in language use (Kemper et al., 2001; Horton et al., 2010).

This work follows several recent studies that used text analytic methods to analyze speech by DJT, including work reporting on dimensions such as analytic thinking (Jordan and Pennebaker, 2017) and communication style (Ahmadian et al., 2017), with a history of studies examining political candidates from their speech (e.g., drawing associations with personality characteristics; Slatcher et al., 2007).

## Materials and Methods

We analyzed speech samples of DJT over the course of 7 years, spanning 2011 to 2017. Interviews were located by browsing all entries in the Trump Archive^1^ and searching for “Trump” in Factbase^2^ (type: interview). In order to maintain consistency across speech samples, we restricted transcripts to unscripted responses to interviews. Any non-spontaneous speech, such as prepared statements (often co-written) and interviewer speech was removed before analysis. Anticipating possible changes in speech across different interviewers and outlets, we further restricted our transcripts to interviews on current affairs on Fox News. To ensure our lexical measures were based on a robust sample of speech, only interviews with at least 1,000 words of speech by DJT were eligible. Because we could only draw on videos made available online, this was not a completely random sample of interviews (i.e., if an interview was not available, we cannot analyze its speech), but we believe the above inclusion criteria limits any potential biases based on availability.

Interviews were transcribed for every month that was available in Factbase and the Trump Archive. If multiple interviews were available for a given month, one was randomly selected for inclusion (to avoid biasing the sample toward later years when interviews became more prevalent). The number of eligible interviews varied by year because DJT was interviewed frequently in some years, but infrequently in others. If a year contained fewer than five eligible transcripts (2012–2014), additional interviews were randomly added from the Fox News website ^3^ (under Politics; searching for “Trump”) until the minimum of five was reached. We note that the practical necessity of having a different number of transcripts per year is not a problem because we use time (i.e., month of interview) as a predicting variable. In addition to yielding a practical minimum number per year, our approach leads to a total (48 transcripts) that is similar to Berisha et al. (2015), aiding a direct comparison to those findings. The study was determined to not fall under human subjects research (according to federal regulations [§45 CFR 46.102(f)]) by the University of Pittsburgh Institutional Review Board.

We followed prior work by statistically analyzing variables that have been sensitive to change in the unscripted speech of a former president (Berisha et al., 2015): count of filler words (“well,” “so,” “basically,” “actually,” “literally,” “um,” “ah/uh”) and NS nouns (nouns and pronouns including the word “thing”), and counts of unique words. Lexical measures can be affected by transcript length (Le et al., 2011) so analyses were restricted to the first 1,000 words (by DJT) of every transcript. We followed the approach taken by Berisha et al. (2015) by stemming all words to their roots using the Lancaster Stemmer in the Natural Language Processing Tool Kit (Bird, 2006) before analysis. As in Berisha et al. (2015), linear regressions were conducted to test if each variable changed over time. Transcript month (i.e., time) served as a predictor for each linguistic variable, giving a statistical test for whether each variable systematically changed with advancing months.

## Results

**FIGURE 1:**
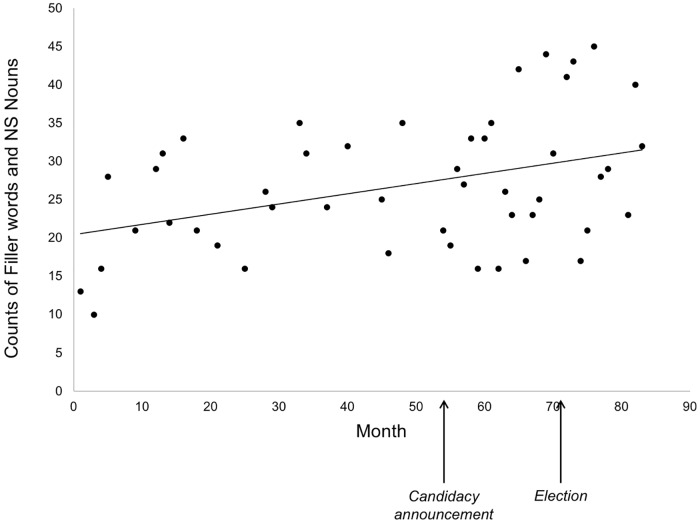
Number of fillers and NS nouns in unscripted interview discourse by Donald J. Trump over time. All transcripts are from the first 1,000 words of unscripted interviews between January 2011 (month 1) and November 2017 (month 83). Months of official candidacy announcement and election are marked.

For the first measure, the use of filler words and NS nouns, DJT showed a significant increase by month, from 2011 to 2017 [*R*^2^ = 0.15, *F*(1,46) = 8.41, *p* = 0.006; *r*(46) = 0.39, mean (*M*) per transcript = 26.83, standard deviation (*SD*) = 8.62)] (Figure 1). Breaking this into components showed an increasing use of filler words [*R*^2^ = 0.11, *F*(1,46) = 5.58, *p* = 0.02; *r*(46) = 0.33, *M* = 19.54, *SD* = 8.11] rather than NS nouns [*R*^2^ = 0.04, *F*(1,46) = 1.94, *p* = 0.17; *r*(46) = 0.20, *M* = 7.29, *SD* = 3.60]. Because running for or taking office could lead to changes in language-use (e.g., as a strategy for media interaction, or through increased stress), we examined whether change was present prior to running for office. We observed a significant increase in filler words and NS nouns in interviews conducted before a formal candidacy announcement for the 2016 Presidential race [in June 2015; month 54 in Figure 1; *R*^2^ = 0.20, *F*(1,19) = 4.68, *p* = 0.04; *r*(19) = 0.44, *M* = 24.24, *SD* = 7.27] suggesting that candidacy or office-related factors do not account for the increase. The trend was not significantly different after the candidacy announcement (*Z* = 0.54, *p* = 0.59), though this period did not reach significance [*R*^2^ = 0.09, *F*(1,25) = 2.47, *p* = 0.13; *r*(25) = 0.30, *M* = 28.85, *SD* = 9.16].

**FIGURE 2:**
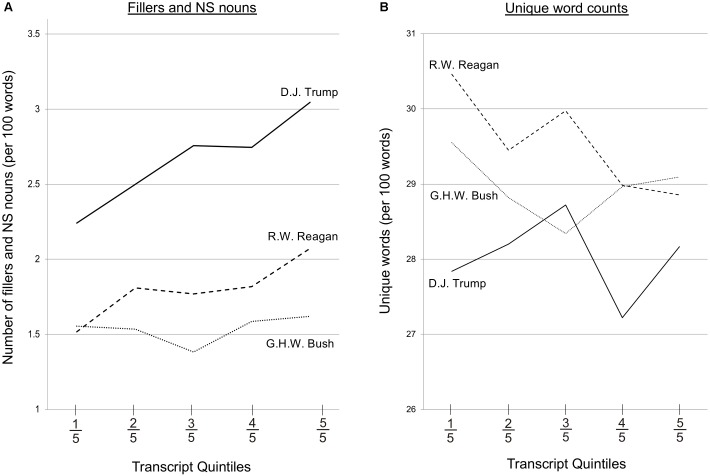
Counts of fillers and non-specific nouns **(A)** and unique words **(B)** used by Presidents Donald J. Trump, Ronald W. Reagan, and George H.W. Bush. Data for R.W. Reagan and G.H.W. Bush are from Berisha et al. (2015). Counts are scaled to every 100 words (i.e., rate), and come from transcripts of unscripted responses to questions from the press with 1,400 (R.W. Reagan and G.H.W. Bush) and 1,000 (DJT) words. As each President has differing numbers of transcripts available, plotted values are averages from each fifth of the transcript set. Note that because Berisha et al. (2015) reported results for R.W. Reagan and G.H.W. Bush by transcript number (rather than month), the displayed transcript quintiles do not map linearly onto time (i.e., a given quintile can represent different durations). Thus, although the trends can be compared, caution is warranted in directly comparing individual quintiles across presidents. As in Berisha et al. (2015), values more than two standard deviations from the mean were removed for DJT: one above the mean filler and NS noun count, and two below the mean unique word count. For unique word count **(B)**, care should be taken not to over-interpret absolute differences between individuals as DJT transcripts are shorter (1,000 vs. 1,400 words), which can give higher unique-word estimates (i.e., the DJT values are relatively inflated compared to the other Presidents; Le et al., 2011).

How does this change compare to the findings of Berisha et al. (2015) for the speech of Presidents Reagan and Bush? Both DJT and President Reagan’s transcripts covered approximately 7 years of interviews, beginning when DJT and President Bush were both 64 years old and President Reagan was 69. We first matched the variables to those used by Berisha et al. (2015) by relating the filler and NS noun variable to “transcript index” (a sequential index of transcript order; e.g., 1,2,3, etc.) rather than to month. The resulting positive relationship for DJT (*r*_46_ = 0.41, *p* = 0.004) was not significantly different (*Z* = 0.26, *p* = 0.79) from the increase previously observed for President Reagan (*r*_42_ = 0.36, *p* = 0.02; Berisha et al., 2015; Figure 2A) but was greater (*Z* = 2.09, *p* = 0.04) than was found for the speech of President Bush (*r*_95_ = 0.05, *p* = 0.61; Berisha et al., 2015). The number of fillers and NS nouns started at a higher level for DJT (*M* = 2.24 per 100 words for the first fifth of transcripts) than for Presidents Reagan (*M* = 1.52 per 100 words; *t*_17_ = 2.50, p = 0.02) and Bush (*M* = 1.55 per 100 words; *t*_28_ = 3.69, *p* = 0.001).

A second measure, unique word count, did not change over time [*R*^2^ = 0.004, *F*(1,46) = 0.19, *p* = 0.67; *r*(46) = 0.06, *M* = 278.60, *SD* = 14.61]. The degree of change was significantly different to President Reagan (*Z* = 2.34, *p* = 0.02), but not to President Bush (*Z* = 0.66, *p* = 0.51; Figure 2B). We note that although the trends can be compared across individuals, the magnitudes cannot, as the DJT transcripts are shorter (1,000 vs. 1,400 words), which will give higher unique-word estimates (i.e., the DJT values are inflated relative to the other Presidents because more unique words occur in a person’s first thousand words compared to their next thousand; Le et al., 2011).

## Discussion

We report the results of a statistical analysis of unscripted speech of DJT. Interview speech contained a systematic increase in use of filler words, but no change in unique word count. The magnitude of the observed increase is not significantly different from that previously observed for President Reagan, and is significantly greater than in the speech of President G.H.W. Bush. Our finding that linguistic change occurred before DJT formally declared candidacy for the 2016 Presidential race suggests several potential explanations as being unlikely. Stress related to assuming the Presidency or a deliberate verbal strategy adopted for the 2016 Presidential race would not have been present when the change first becomes apparent. There are a number of possible reasons for the observed change. Prior research has associated linguistic change with advanced aging (Kemper et al., 2001; Horton et al., 2010), as well as with the onset of dementia (Snowdon et al., 1996; Le et al., 2011). In this instance, the speech we analyzed first occurred when DJT and President Bush were 64 years old, and when President Reagan was 69. Individuals respond differently to aging, however, so it is not possible to distinguish between the above possible explanations, and identifying the reason for this systematic change falls outside the scope of this study. We further stress that although our findings provide evidence of linguistic change, they should not be used to infer a change in cognitive state: these variables are not validated measures of cognitive change and should not be interpreted as such.

In contrast to filler use, we did not observe a change in unique word count. It is difficult to determine why one measure shows a change while another does not, though we note that our examined word count (1,000 compared to 1,400 words used previously; Berisha et al., 2015) could reduce power to detect change. Another possibility is that any change in unique word count is being masked by greater off-topic speech (Trunk and Abrams, 2009) or use of non-normative words (Kavé et al., 2009), which can both increase with age (and increase unique word count). An important consideration is that the speech samples we analyzed relate to current affairs. The generalizability of the findings to different topics and contexts is therefore unknown. The analysis of additional linguistic variables might shed further light on changes in DJT’s speech.

## Author Contributions

MC conceived and planned the study. MC and JP contributed to the methods, transcription of audio recordings, analyses, interpretations of results, and drafting of the manuscript.

## Conflict of Interest Statement

The authors declare that the research was conducted in the absence of any commercial or financial relationships that could be construed as a potential conflict of interest.

## Footnotes

1 https://archive.org/details/trumparchive

2 https://factba.se/transcript

3 www.foxnews.com
